# Placental Transcriptome Profiling in Subtypes of Diabetic Pregnancies Is Strongly Confounded by Fetal Sex

**DOI:** 10.3390/ijms232315388

**Published:** 2022-12-06

**Authors:** Sarah M. Kedziora, Benedikt Obermayer, Meryam Sugulle, Florian Herse, Kristin Kräker, Nadine Haase, Immaculate M. Langmia, Dominik N. Müller, Anne Cathrine Staff, Dieter Beule, Ralf Dechend

**Affiliations:** 1 Experimental and Clinical Research Center (ECRC), a Joint Cooperation between the Charité—Universitätsmedizin Berlin and Max-Delbrück-Center for Molecular Medicine, 13125 Berlin, Germany; 2 Max-Delbrück-Center for Molecular Medicine in the Helmholtz Association (MDC), 13125 Berlin, Germany; 3 Charité—Universitätsmedizin Berlin, Corporate Member of Freie Universität Berlin, Humboldt Universität zu Berlin, Berlin Institute of Health, 10178 Berlin, Germany; 4 DZHK (German Centre for Cardiovascular Research), Partner Site, 10785 Berlin, Germany; 5 Berlin Institute of Health, Charité—Universitätsmedizin Berlin, Core Unit Bioinformatics, 10117 Berlin, Germany; 6 Faculty of Medicine, University of Oslo, 0372 Oslo, Norway; 7 Division of Obstetrics and Gynaecology, Oslo University Hospital, 0424 Oslo, Norway; 8 HELIOS Clinic, Department of Cardiology and Nephrology, 13125 Berlin, Germany

**Keywords:** diabetes mellitus, human, placenta, pregnancy, RNA sequencing

## Abstract

The placenta is a temporary organ with a unique structure and function to ensure healthy fetal development. Placental dysfunction is involved in pre-eclampsia (PE), fetal growth restriction, preterm birth, and gestational diabetes mellitus (GDM). A diabetic state affects maternal and fetal health and may lead to functional alterations of placental metabolism, inflammation, hypoxia, and weight, amplifying the fetal stress. The placental molecular adaptations to the diabetic environment and the adaptive spatio–temporal consequences to elevated glucose or insulin are largely unknown (2). We aimed to identify gene expression signatures related to the diabetic placental pathology of placentas from women with diabetes mellitus. Human placenta samples (*n* = 77) consisting of healthy controls, women with either gestational diabetes mellitus (GDM), type 1 or type 2 diabetes, and women with GDM, type 1 or type 2 diabetes and superimposed PE were collected. Interestingly, gene expression differences quantified by total RNA sequencing were mainly driven by fetal sex rather than clinical diagnosis. Association of the principal components with a full set of clinical patient data identified fetal sex as the single main explanatory variable. Accordingly, placentas complicated by type 1 and type 2 diabetes showed only few differentially expressed genes, while possible effects of GDM and diabetic pregnancy complicated by PE were not identifiable in this cohort. We conclude that fetal sex has a prominent effect on the placental transcriptome, dominating and confounding gene expression signatures resulting from diabetes mellitus in settings of well-controlled diabetic disease. Our results support the notion of placenta as a sexual dimorphic organ.

## 1. Introduction

The placenta is a temporary mammalian organ with the vital function of fetal nutrient supply and waste removal. Its unique structure, with tissue emerging from both embryonic and maternal origin, is necessary for healthy fetal development [1]. The rapid placental growth includes proliferation, differentiation and invasion of trophoblasts, implantation, remodeling, and angiogenesis, all of which are decisive factors for healthy pregnancies and outcomes [2]. Placental maldevelopment and dysfunction are involved in the major, often co-occurring obstetric syndromes, including preeclampsia (PE), fetal growth restriction, preterm birth and gestational diabetes mellitus (GDM) [3,4,5]. 

In a diabetic environment, a variety of structural and functional changes take place in the placenta. Besides heavier placentas, inflammation and DNA methylation, altered expression of genes regulating for growth, glucose metabolism, cytoskeletal structure, oxidative stress and apoptosis have been described [6,7]. The extent of structural and functional changes depends on multiple variables, including the type of diabetes mellitus (DM) and glycemic control [6]. 

DM can be present before, and throughout, gestation as in DM type 1 (DM1) or type 2 (DM2). In both cases, placentation takes places under the complex diabetic milieu, although the pathophysiology of DM1 and DM2 differs. During the first trimester, the placental growth rate is highest and the placenta is therefore extremely sensitive to environmental influences [6]. Secondly, DM can occur during gestation (gestational diabetes mellitus; GDM) and resolve postpartum in most cases [8]. In women with GDM, impaired glucose tolerance develops and leads to more short-term molecular alterations [2]. 

Both pregestational and gestational diabetes imply a well-described and significantly increased risk of adverse maternal and offspring pregnancy outcomes, as well as long-term diseases such as obesity and cardiovascular disease [5,9]. Central to short-term association is the placental pathology, most likely due to maternal metabolic and inflammatory changes in a diabetic milieu. The long-term consequences are likely explained by in utero programming impacting on health or disease later in life, independent of the DNA sequences that are inherited in a person’s genetic code (i.e., developmental origins of health and disease) [10]. 

One potential complication to pregestational and gestational diabetes during pregnancy is PE [11], which is also closely linked to a dysfunctional placenta [6]. PE superimposed on a diabetic condition is a major risk factor for preterm delivery, maternal and offspring morbidity and future health [3,12]. 

The precise mechanisms by which pregestational or gestational diabetes contribute to placental dysfunction are unknown [1,6]. Besides the effects of glucose and insulin on the placenta, reactive oxygen species production in first trimester trophoblasts, insulin resistance of trophoblasts, and altered oxygen tension in the intervillous space have been addressed [13]. It is likely that pregestational and gestational diabetes may have different adaptation mechanisms, including differential effects on placentation, as GDM is usually not present during the most important placentation period [14]. 

The aim of this study is to unravel novel pathways related to the contribution of diabetes to pregnancy pathologies, including diabetic pregnancies complicated by PE, by transcriptome analysis of placenta tissues. Various studies suggest that placental inflammatory pathways, stress response and gene expression patterns are related to maternal pregnancy complications. Understanding the contribution of the placental transcriptome profile to placental differences responsible for fetal and maternal health—such as fetal growth, preterm birth and survival—is essential. By gene expression analysis, we aimed to identify differentially expressed genes (DEGs) and associated signaling pathways among GDM, pregestational DM, or diabetic pregnancies complicated with PE. Here we present, to our knowledge, the first RNA sequencing dataset of the placental transcriptome from women with GDM, DM1, or DM2, and in DM pregnancies with superimposed PE, to gain a profound understanding of transcriptional placental profiles during the various forms of a diabetic pregnancy.

## 2. Results

### 2.1. Clinical Characteristics of the Study Cohort

The study cohort consisted of healthy and diabetic women, while other diabetic patients developed superimposed PE during pregnancy. Clinical characteristics of these healthy and diabetic pregnant women (subgroups) are shown in Table 1. Characteristics of diabetic women with a pregnancy complicated with PE are shown in Table 2. Further, the characteristics of all women with the different types of DM or DM superimposed with PE are shown in Table 3. The BMI of women with GDM and DM2 was higher at the beginning of pregnancy and at delivery (GDM: 33.2 ± 6.77; DM2: 38.57 ± 2.89; Table 1) compared to healthy CTL (CTL: 28.79 ± 3.98), even when complicated with PE (Table 2). The diabetic pregnant women superimposed with PE not only developed hypertension (Table 2), but also proteinuria (Table 3) during pregnancy. Early-onset PE appeared in 37.5% of pregnant women. HbA1c was higher during pregnancy in pregestational diabetic women compared to women who developed GDM, independent of PE. All subgroups of women with DM without superimposed PE in pregnancy had, as expected, heavier babies and higher birthweight percentiles compared to CTL (Table 1). Pregnancies with DM superimposed with PE had, as expected, an earlier delivery than CTL (Table 3). Women with GDM + PE had consequently lower birth weight and smaller newborn weight percentiles than CTL, a proxy of placental dysfunction. While DM1 + PE had higher birthweight percentiles but similar birthweights as CTL, the birthweight and newborn weight percentile were not different in pregnancies of DM2 + PE compared to CTL (Table 2).

### 2.2. Placentas of Patients with Diabetes Reveal Differentially Expressed Genes

**Figure 1:**
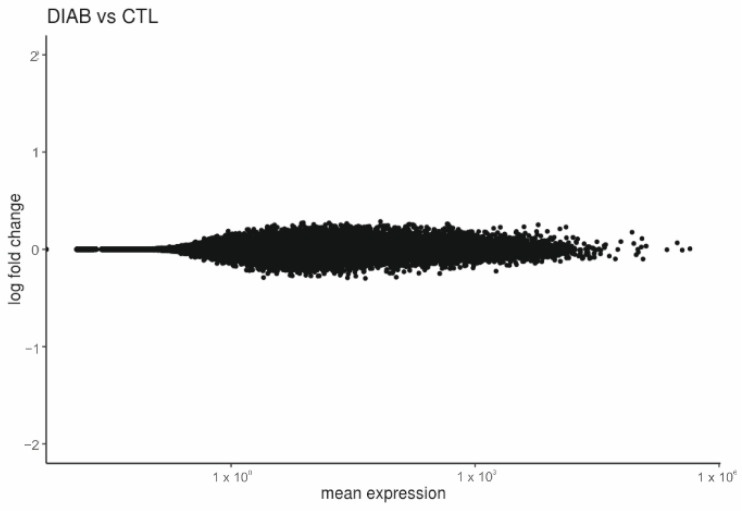
The effect of diabetes on the placental transcriptome in comparison to healthy controls. MA-plot showing the log2-fold change over mean expression for all genes for the diabetes group (DIAB) without PE (*n* = 32) versus CTL (*n* = 29). DIAB = patients with DM1, DM2 and GDM; CTL = healthy controls.

The gene expression between diabetic and healthy CTL placentas using DESeq2 [15] with fetal sex as covariate did not show any DEGs at adj. *p*-value < 0.05 (see MA-plot in Figure 1). 

**Figure 2:**
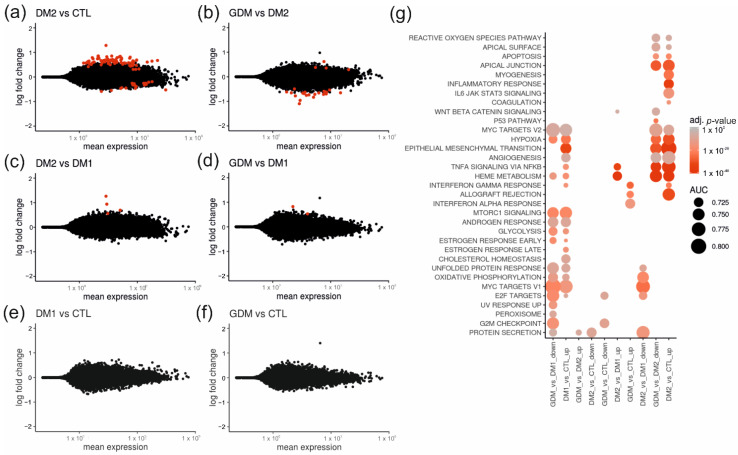
Diabetes subgroups show an altered placental transcriptome in comparison to healthy controls. (**a**–**f**) MA-plots showing log2-fold change as a function of mean expression for the diabetes subgroups DM1 (*n* = 17), DM2 (*n* = 3), GDM (*n* = 12) versus CTL (*n* = 29). DEGs (adj. *p*-value < 0.05) are marked in red. (**g**) Gene-set enrichment analysis with tmod for up- and down-regulated genes in these contrasts. The adjusted *p*-value is color-coded and the AUC statistic is displayed as the dot size. GDM = gestational diabetes mellitus; DM1 = type I diabetes mellitus; DM2 = type II diabetes mellitus; CTL = healthy controls.

Subsequently we analyzed the diabetic subgroups and found relatively few significant gene expression differences between placentas from DM1 (*n* = 17), DM2 (*n* = 3), GDM (*n* = 12) and CTL (*n* = 29) patients (Figure 2). Most DEGs (75 up-regulated and 18 down-regulated at adj. *p*-value < 0.05) were observed when comparing DM2 to CTL (Figure 2a), summarized in Supplementary Table S1. Figure 2b shows the comparison between DM2 and GDM, where we identified four up- and 23 down-regulated genes (Supplementary Table S1). Pathway analysis showed that several DEGs in the comparison of DM2 with either CTL or GDM were associated with metabolism (such as *SELENBP1* [16], *CNNM* [17], and *SOXS3* [18]) and placental metabolism (such as *ACSL6* [19] and *ARID5A* [20]). DM1 versus DM2 (Figure 2c) showed 4 up-regulated genes (Supplementary Table S1), and GDM versus DM1 showed only two up-regulated genes (*SMCO3* and *NQO1*), both linked to DM [21,22] (Figure 2d). In contrast, DM1 versus CTL (Figure 2e) and GDM versus CTL (Figure 2f) did not show significant DEGs. Our results show that gene expression differences in DM1 and DM2 showed similar patterns to each other (Pearson’s R = 0.47 between log2-fold changes), whereas GDM was more similar to CTL.

Since only few informative DEGs were observed in the analysis of 77 placental transcriptomes, we next conducted gene-set enrichment analysis using HALLMARK transcriptional gene sets on the estimated log2-fold changes for all genes in the comparisons of different diabetic conditions in pregnancy (Figure 2g). Several gene sets were altered but no uniform dysregulated pathway between the different subtypes of diabetic pregnancy and CTL was observed. Genes involved in the epithelial–mesenchymal transition, hypoxia, angiogenesis or inflammation were consistently altered when comparing the placental transcriptome of DM2 versus GDM or CTL. DM1 versus GDM or CTL showed similar patterns, including in pathways involved in unfolded protein response, oxidative phosphorylation, and proliferation, but the differences were less pronounced. 

**Figure 3:**
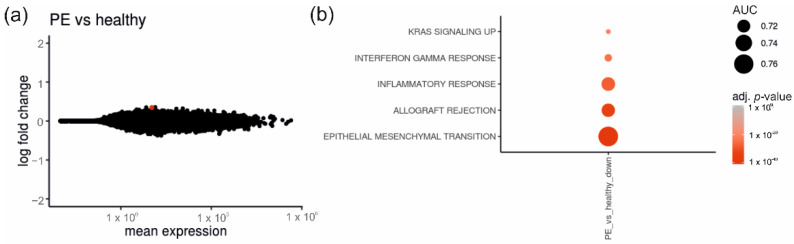
The effect of diabetes superimposed with preeclampsia on the placental transcriptome in comparison to healthy controls. (**a**) MA-plot shows log2-fold change over mean expression for all diabetic placenta samples superimposed with PE (*n* = 16) versus CTL (*n* = 29). DEGs are highlighted in red. (**b**) The gene-set enrichment analysis displays significantly altered gene sets in this comparison. PE = includes patients with DM1 + PE, DM2 + PE and GDM + PE; CTL = healthy controls.

When investigating the data of placentas from diabetic pregnancies complicated by PE (*n* = 16) in comparison to CTL (*n* = 29), we observed a similar pattern. The comparison of PE against CTL gave only one DEG (Figure 3a, Supplementary Table S1) and gene-set enrichment analysis mainly showed alterations of genes involved in the epithelial–mesenchymal transition and inflammatory response (Figure 3b).

### 2.3. Placental RNA Sequencing Samples Group Mainly According to Fetal Sex

**Figure 4:**
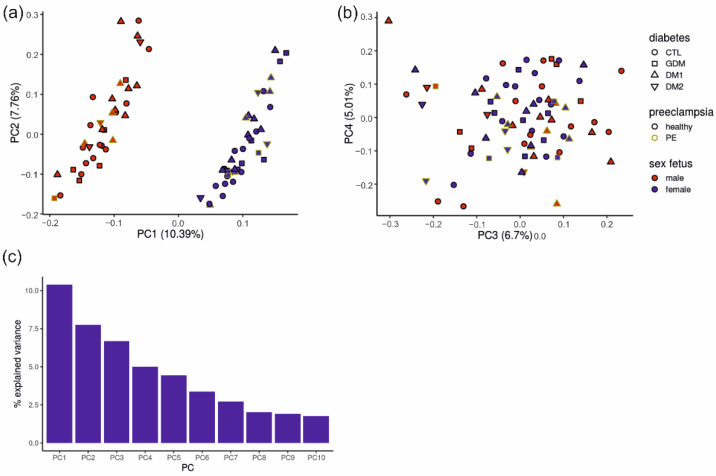
Placental samples group mainly due to fetal sex. (**a**) Principal component analysis (PCA) displays the highest amount of explained variance (10.39%) between samples in PC1 and the second highest (7.76%) in PC2. Placental samples cluster according to the fetal sex. (**b**) PC4 and PC3 do not cluster subjects into groups in the PCA. Black border: CTL (*n* = 29, circle); GDM (*n* = 12, square); DM1 (*n* = 17, triangle pointed top); DM2 (*n* = 3, triangle pointed bottom). Green border: GDM + PE (*n* = 4, square); DM1 + PE (*n* = 8, triangle pointed top); DM2 + PE (*n* = 4, triangle pointed bottom). Fetal sex is indicated in blue = male and red = female. (**c**) Percentage of explained variance by each PC from PC1 to PC10. Diabetes types are indicated with symbols. CTL = healthy control; GDM = gestational diabetes mellitus; DM1 = Diabetes mellitus type 1; DM2 = diabetes mellitus type 2.

We next performed principal component analysis (PCA) to identify what factors could confound the differences between the clinical subgroups. In the first principal component with almost 11% explained variance, the data set clustered into two groups according to the fetal sex (Figure 4a), driven by sex-specific genes such as *XIST*, *UTY*, *USP9Y*, *DDX3Y* and *KDM5D*. The other PCA components were not clearly related to clinical parameters or driven by systematic gene groups. The second PC (7.76% explained variance, driven by *TAC3*, *AADACL3*, *DIO2*, *NOTUM* and *HTRA4*) showed an even distribution of samples without obvious clustering according to the clinical diagnosis (Figure 4a). Similarly, other components contributed little to the explained variance (Figure 4b) and did not induce a clustering of samples.

### 2.4. Of All Gene Sets and Clinical Data, the Fetal Sex and Diagnosis Contribute Most to Principal Components 

**Figure 5:**
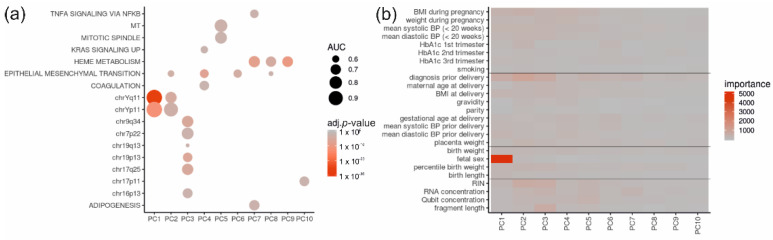
Association of principal components to Y-chromosome linked genes and fetal sex. (**a**) Gene-set enrichment analysis shows how principal components (PC) are associated with particular gene sets. PC1 and PC2 are strongly influenced by Y-chromosome genes. The effect size (AUC) is shown as dot size and the color indicates significance (adjusted *p*-value). (**b**) The heat map shows the contribution of clinical (meta data) and quality control parameters to the PCs. The impact (“importance”) is color-coded with low contribution in grey and high contribution in red. Fetal sex clearly contributes to PC1. The figure is based on all CTL (*n* = 29) and diabetic placenta samples without PE (*n* = 32).

We next analyzed which gene sets contributed most to the PCA and used gene-set enrichment analysis of the gene loadings. PC 1 and, to a lesser extent, PC 2 were strongly associated with Y-chromosomal genes. Higher components showed much weaker association with chromosomal locations or functions such as epithelial–mesenchymal transition, mitochondria, cell cycle or heme-metabolism (Figure 5a). 

As none of the gene sets convincingly explained the variation in PC2 and below, we next investigated whether PCs were associated with clinical data (meta data) or technical quality-control parameters. We used random forest regression to determine the contribution of each clinical parameter to a particular PC. Again, fetal sex contributed overwhelmingly to the first PC, while the diagnosis prior to delivery and other parameters, such as BMI, Hba1c, blood pressure during pregnancy as well as RNA quality (RIN value), contributed much less to the PCs (Figure 5b). 

### 2.5. Comparison between Placentas of Male or Female Fetus Display Several DEGs

**Figure 6:**
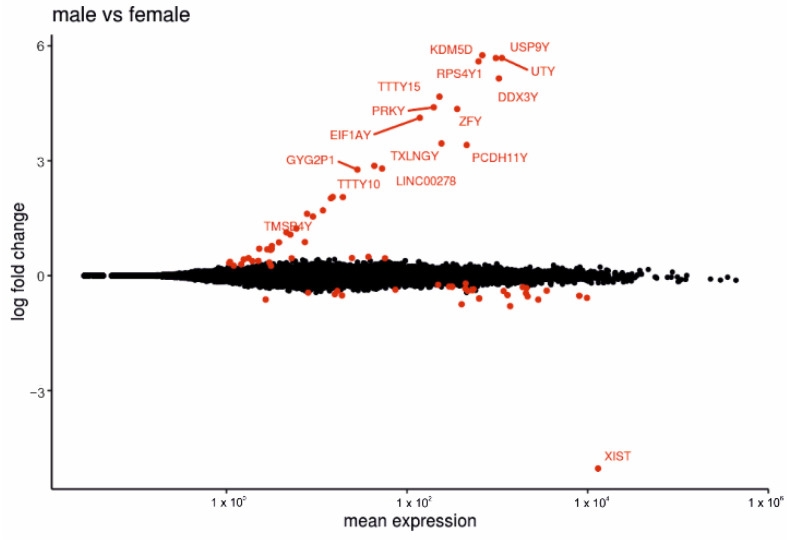
Fetal sex effect on gene expression in placenta. The MA-plot displays the effect of fetal sex on gene expression in placentas with male (*n* = 36) or female fetuses (*n* = 41). Differential genes (adj. *p*-value < 0.05) are marked in red. Positive log2-fold change indicates male-specific expression; negative log2-fold changes indicate female-specific expression.

We next performed differential expression analysis between placentas from female fetus (*n* = 41) and male fetus (*n* = 36) pregnancies with clinical diagnoses (diabetes subtypes and PE) as covariates. The comparison revealed 78 up-regulated and 76 down-regulated genes (Supplementary Table S2) that are highlighted in the MA-plot (Figure 6). Genes with increased expression in placentas with male fetuses (e.g., *DDX3Y*, *ZFY*, *KDM5D* and *UTY*) were mainly located on the Y-chromosome, but other DEGs such as *CTFR*, *SPP1* and *ZNF711* were located on the X-chromosome or autosomal chromosomes. 

The 76 genes with higher expression in placentas with female fetuses were mostly located on the X-chromosome; among them *XIST*, *FTX*, *ZFX*, *SMC1A*, *STS* and *FMR1*. Some DEGs were not located on the X-chromosome but were associated with placentation. For example, *PLAU* has been associated with trophoblast invasion [23] and *SWAP70* has been associated with placentation [24]. We performed analyses of up-regulated and down-regulated genes from the comparison of male and female placenta samples. There were no KEGG terms associated with the gene sets. In addition, no relevant gene ontology terms were present.

Finally, we compared our results to a study by Gonzales et al. on sex differences in the late first trimester in the human placenta transcriptome (GSE109120) [25] and found a good overlap: 33 male and 15 female genes were significantly different in both studies; six male and two female genes were discovered only in our data; and 13 male and 11 female genes were detected only in their study.

## 3. Discussion

In the present study, we show that the placental transcriptome signature from healthy CTL is similar to pregnancies complicated by DM. When diabetic pregnancy was complicated with PE, again only mild differences in the placental transcriptome were observed compared to healthy CTL. At the transcriptome level, the placentas from women with GDM were more similar to CTL than DM1 or DM2, while the latter two were relatively similar to each other. Genes linked to pregnancy complications or metabolic diseases contributed little to the observed variance, and no defined gene set or pathway could be directly attributed as the main contributor to the observed differences. The analysis of clinical data showed only minor contribution of the clinical diagnosis to the variances between diabetic subgroups. Remarkably, we identified fetal sex to be the strongest contributor to differences in the transcriptome profile. Our study has two unexpected results, which warrant further studies and follow up. Firstly, even though the placenta is heavily affected by the various forms of DM and by superimposing PE, the transcriptome profile appears to be only marginally altered. However, the transcriptome does neither fully reflect the profound metabolic and pro-inflammatory alterations in the pregnant mother, nor does it fully explain the adverse maternal or fetal morbidity and mortality. Our second remarkable finding is that fetal sex has a profound influence on the placental transcriptome, indicating that sex-specific alterations in placental function are more important than previously expected and supports that the sexual dimorphism of the placenta should not be ignored in scientific practice. 

RNAseq is a powerful method to quantify transcriptomes [26]. It allows identification of pathologic alterations that are linked to clinical diagnosis, which can help to develop new biomarkers for future prediction, diagnosis, and therapy.

Several pregnancy complications have been linked to altered gene expression via studies on specific genes and transcriptome analysis using microarray or RNAseq [27,28,29]. Sõber et al. highlighted differences in gene expression pattern with RNAseq analysis (*n* = 8/group) of placentas from women with PE, while GDM or small- and large-for-gestational age showed less intensive expression differences [30]. Although the sample number was relatively small, the researchers found that the GDM placenta transcriptome differed the least from healthy CTL placentas, while placentas from preeclamptic women showed the strongest differences in gene expression pattern, followed by those small- or large-for-gestational-age. Lekva et al. did not find altered genes in the transcriptome profile of placentas from women with GDM, and only five DEGs in term placentas from women with PE [31]. The observations on GDM placental transcriptome of Sõber et al. and Lekva et al., along with our findings, suggest that gene expression in the placenta is not sufficiently altered during a diabetic pregnancy to produce observable effects beyond inter-individual variability, possibly due to well-treated disease. In our study cohort, 35.7% of women with GDM were treated with insulin and/or metformin (Table 1 and Table 2). The lack of an effect on the placental transcriptome could result from relatively mild BMI at delivery (GMD: 33.2; GMD + PE: 38.5) in our study, which serves as an indicator for morbid obesity, and is related to inflammation and dysregulated placental function [32].

Multiple other studies highlight the transcriptome profile of placentas from women with GDM compared to controls. While two microarray studies identified seven [33] and 66 [34] DEGs, respectively, which were associated with apoptosis and inflammation, another RNAseq study found 281 DEGs [35]. Since GDM is a time-restricted disease during pregnancy (although the risk of DM2 is increased long-term), the question arises as to whether the placental transcriptome reflects the metabolic changes of that period. This question is so far unresolved, as some studies found no or only minor changes in placental gene expression [30,31] when comparing GDM to healthy controls, while other studies identified DEG patterns in placentas of women with GDM and assigned dysregulated genes to pathways of glucose metabolism and immunology [36,37,38]. 

Only few studies have considered pregestational diabetes subtypes. The published analyses include small sample numbers (*n* = 3 and *n* = 6, respectively), which makes expression differences difficult to be identified [39,40]. 

Limiting to our study design is the relatively small sample size in some subgroups, which is attributed to the limited number of women suffering from multiple pregnancy-related complications. Therefore, careful interpretation of the data is necessary. Nonetheless, the variety of subgroups characterized gives a rare insight into the placenta transcriptome and results should be judged wisely. These limitations go along with a low number of studies focusing on transcriptome analysis of placentas from women with DM2 because of limited numbers of pregnant women suffering from DM2 [41]. In one study, placentas from women with GDM (*n* = 14) and DM2 (*n* = 3) were analyzed by RNAseq and DNA methylation [39]. The authors report differences in methylation and transcriptome level in placentas from male and female offspring to be more pronounced than the difference between clinical diagnosis. In our transcriptome analysis, placentas from women with DM2 showed the most distinct pattern compared to both healthy CTL placentas and placentas from women with GDM, however the number was also small. These findings are reasonable as the placenta is exposed to a diabetic surrounding. While some studies focus on the analysis of pre-selected genes in placenta tissues from women with DM1 [42,43], global transcriptome analysis is lacking. 

In contrast to our results, other studies have identified multiple DEGs in placentas from women with PE, yet these women did not have additionally diagnosed DM [44]. Buckberry et al. identified gene sets in the human placenta that were preserved at different timepoints of gestation, with altered expression patterns in placenta samples from women with PE [45]. In another study with previously identified gene expression-based PE subtypes, the severity of histopathological placental lesions matched the PE subtypes [46].

Although various studies suggest that placental inflammatory pathways, stress response and gene expression patterns are related to maternal pregnancy complications, we could not strengthen these observations with our study [38,47,48]. In our RNAseq data, fetal sex contributed most to the observed transcriptome pattern. The impact of fetal sex on the placental transcriptome had previously been observed in a microarray analysis study. Placentas from women with PE complicated with either HELLP (= hemolysis, elevated liver enzymes, low platelet), IUGR (= intrauterine growth restriction) or SGR (= small for gestational age), showed DEG patterns that varied due to fetal sex [49]. A RNAseq analysis of first trimester human placentas from healthy women highlighted early differences in the transcriptome with 58 DEGs between placentas from female and male fetuses [25]. Gonzales et al. identified genes located on Chromosome 19 contributing most to DEGs, followed by genes on the Y-chromosome [25]. Also, in our data set, some DEGs were located on Chromosome 19, while a larger number were located on gonosomes. Besides X-chromosomal-linked genes, Sood et al. detected some autosomal genes, suggesting that the difference in expression might be due to underlying differences in male and female physiology [50]. Another study analyzed placental transcriptomes of women with PE or fetal growth restriction using RNAseq; this found strong placental transcriptome clustering according to fetal sex and identified sex-biased pathways [51]. The meta-analysis of microarray data highlighted 88 autosomal genes that were differentially expressed between placentas bearing a male or female fetus [45]. Altogether, these observations are in agreement with our findings and verify that the fetal sex strongly contributes to the placental transcriptome profile. 

Our findings comply with other specific features related to fetal sex. Sex-specific placental differences are relevant for fetal growth, preterm birth, and survival [52]. In addition, sex-specific alterations of gene expression have not only been reported in genes located on either the X- or Y-chromosome, but also on autosomal genes that encode immune and hormonal pathways [52].

Our data contribute to the concept of the placenta as a sexual dimorphic organ. It also suggests that the transcriptional signature of the placenta is not very informative for understanding maternal–placental–fetal health in the context of well-treated diabetic pregnancies and superimposed PE. Our data is in line with previous findings that emphasize the important influence of fetal sex on the placental transcriptome. In our analysis, the effect on variance by fetal sex is stronger than the clinical diagnosis. Thus, we feel confident to advise all researchers who aim to investigate the placental transcriptome profiles not only to adjust for fetal sex, but to consider fetal sex in their experimental planning, including for sample size calculation.

In summary, our data underlines the concept that the placenta is a sexual dimorphic organ with gonosomal genes strongly contributing to the transcriptome signature of the placenta. Future studies are needed to clarify which adjustments to a pathological pregnancy are sex-specific and which are not. Furthermore, the role of epigenetic alterations in the placenta as the result of exposure to the diabetic milieu in pregnancy should be explored.

## 4. Materials and Methods

### 4.1. Study Population and Sample Collection

The placenta samples were collected between 2001–2013 at the Oslo University Hospital, Norway as a part of the Oslo Pregnancy Biobank. The study was approved by the Regional Committee of Medical Research Ethics in South East Norway (Oslo Pregnancy Biobank REK: 2010/1850/REK South East C). The population used in this study includes 77 placenta samples from women with either healthy or complicated pregnancy. The set consists of 29 healthy controls (CTL), 12 women with gestational diabetes (GDM), 17 women with type I diabetes mellitus (DM1) and three women with type II diabetes mellitus (DM2). The DM patients were grouped according to the World Health Organization criteria [53,54]. PE was diagnosed on the basis of new-onset hypertension (>140/90 mmHg) and proteinuria during pregnancy [55]. A written informed consent was provided by all patients. 

The placenta was delivered following caesarean section as previously described [56,57]. Briefly, following the delivery of the baby, 3–5 IU oxytocin was given to the mother intravenously. The placenta was separated spontaneously from the uterine wall and gently removed. Placental plus umbilical cord weight was noted. The placental villous biopsies were taken from macroscopically normal-appearing cotyledons, avoiding the decidual layer as previously described [54,58]. After collection, tissue samples were immediately frozen and stored at −80 °C until further analyzed. Blood sample biochemistry, blood pressure and BMI were analyzed as previously described [59]. The newborn weight percentiles were calculated according to Norwegian fetal growth curves as previously described [60]. 

### 4.2. RNA Isolation

RNA was isolated from 77 placenta samples using Qiagen RNeasy Kit (Qiagen). After homogenization of the placental tissue sample, the RNA extraction was performed following the manufacturer’s protocol. Only 160 µL of the watery phase was combined with an equal amount of 70% ethanol and loaded onto the RNeasy Mini Column. The RNA was eluted with 40 µL RNase-free water. The RNA concentration, size range and quality were measured using Agilent Bioanalyser 2100, Eukaryote Total RNA Nano Series II according to the manufacturer’s protocol (Agilent RNA 6000 Nano Kit Guide). A Qubit Fluorometric Quantitation Assay was used to validate RNA concentration. Samples used for RNA sequencing data analysis had a mean RIN of 5.1 (±1.09 SD).

### 4.3. RNA Sequencing

The Illumina TruSeq stranded total RNA Library Prep Kit was used for library preparation and RNA samples were diluted in water to 1000 ng/µL. The sequencing was done on the Illumina HiSeq4000 system at the Scientific Genomics Platforms at the Max Delbrück Centre for Molecular Medicine, Berlin. A loading concentration of 200 pM, paired-end run-type mode and a read length of 75 bp was used. 

### 4.4. Sequencing Data Processing

Sequencing reads were aligned to the human genome (GRCh38) using STAR (v2.6.1a, Dobin et al., USA) [61]. Gene expression was quantified using featureCounts (v1.6.3, online available at www.bioconductor.org, Liao et al., USA) [62] and the Gencode v25 reference, including non-coding genes. We then used DESeq2 (v1.18.1, online available at www.bioconductor.org, Love et al., USA, DE) [15] to detect differentially expressed genes for comparisons between groups (DM/CTL/PE) and subgroups (DM1/DM2/GDM with and without PE), using fetal sex as a covariate. Gene-set enrichment on estimated log2-fold change values was performed using tmod [63] and Hallmark gene sets from MSigDB (version 7, Broad institute, San Diego, CA, USA). For the principal component analysis (PCA) we used regularized log2-transformed counts for the top 5000 variable genes. Gene-set enrichment on PC gene scores was performed using tmod [63] and Hallmark and positional gene sets from MSigDB (version 7, Broad institute, San Diego, CA, USA). We used random forest regression with the randomForest package v4.7-14 [64] to infer the contribution of each clinical parameter to the principal components of the analysis, imputing missing values with the roughfix method. 

### 4.5. Statistics

The sequencing data was statistically analyzed using R software (v3.4.4, R core team, online available www.r-project.org), SPSS (v1.2.0, IBM, USA), GraphPad Prism (v6, GraphPad Software, US) and Microsoft Excel (v2211, Microsoft 365, USA). Clinical parameters are displayed as mean ± standard deviation or a percentage. Group differences were tested with one-way ANOVA with Sidak’s multiple comparisons, adjusted *p*-values are indicated and significant when *p* < 0.05.

## Figures and Tables

**Table 1:** Clinical characteristics of study group participants for diabetic subgroups without PE (*n* = 61, including 29 CTL and 32 DM).

Characteristics	CTL (*n* = 29)	GDM (*n* = 12)	*p*-Value CTL vs. GDM	DM1 (*n* = 17)	*p*-Value CTL vs. DM1	DM2 (*n* = 3)	*p*-Value CTL vs. DM2
**maternal characteristics <20 weeks gestation**							
BMI	23.68 ± 4.06	28.03 ± 5.65	0.02	25.22 ± 3.95	0.81	34.58 ± 1.20	0.00
SBP, mmHg	111.03 ± 9.41	113.08 ± 9.27	0.99	113.81 ± 8.42 (16)	0.98	127.67 ± 19.14	0.15
DBP, mmHg	66.21 ± 7.04	70.42 ± 7.35	0.48	70.06 ± 5.92 (16)	0.48	75 ± 1.73	0.29
HbA1c 2. Trimester, %	NA	5.2 ± 0.62 (4)	NA	6.17 ± 0.62 (15)	NA	5.87 ± 0.09	NA
smoking, % (*n*)	13.79 (4)	16.67 (2)	NA	5.88 (1)	NA	33.3 (1)	NA
height, m	1.70 ± 0.06	1.67 ± 0.06	0.87	1.67 ± 0.05	0.75	1.68 ± 0.05	0.99
weight, kg	68.17 ± 11.96	78.34 ± 14.77	0.13	70.53 ± 11.48	0.99	98 ± 5.29	0.00
**maternal characteristics at delivery**							
BMI at delivery	28.79 ± 3.98	33.2 ± 6.77 (11)	0.05	29.7 ± 4.03	0.99	38.57 ± 2.89	0.00
pre-operative SBP, mmHg	124.42 ± 12.22 (26)	132 ± 10.89 (9)	0.72	138.93 ± 14.35 (15)	0.02	144.5 ± 0.70 (2)	0.35
pre-operative DBP, mmHg	72.96 ± 10.78 (26)	80.44 ± 15.48 (9)	0.42	79.13 ± 9.95 (15)	0.44	73.0 ± 2.83 (2)	>0.99
proteinuria, % (*n*)	4 (1)	10 (1)	NA	13,32 (1)	NA	0 (0)	NA
HbA1c 3. Trimester, %	NA	5.79 ± 0.55 (11)	NA	6.27 ± 0.57 (15)	NA	6.2 ± 0.22	NA
medication, % (*n*) [insulin, metformin]	0 (0)	41.67 (5)	NA	88.24 (15)	NA	100 (3)	NA
gestational age, days	271.93 ± 7.94	267.67 ± 13.03	0.94	260.65 ± 17.19	0.06	269.67 ± 4.16	>0.99
age, years	32.66± 4.50	34.58 ± 3.61	0.77	33.59 ± 3.76	0.98	31.0 ± 6.56	0.99
parity, count	0.93 ± 0.88	1.08 ± 0.90	0.99	0.71 ± 0.59	0.93	0.33 ± 0.58	0.78
gravidity, count	2.72 ± 1.25	3.25 ± 1.42	0.77	2.29 ± 1.16	0.83	2.00 ± 1.00	0.91
blood sugar, mmol/l	3.98 ± 0.48 (9)	4.78 ± 1.05 (9)	0.72	6.57 ± 1.56 (13)	0.00	6.53 ± 1.45	0.03
**birth outcome**							
fetal sex, female/male, count	15/14	7/5	NA	8/9	NA	1/2	NA
birth weight, g	3342 ± 450	3697 ± 844	0.72	3611 ± 864 (16)	0.85	4515 ± 788	0.09
birth length, cm	50.05 ± 1.49 (21)	50.55 ± 3.55 (10)	0.99	49.57 ± 4.09 (14)	0.99	53.67 ± 2.89	0.34
percentile birthweight	51.04 ± 27.68	73.18 ± 33.06	0.22	78.87 ± 30.69 (16)	0.03	97.19 ± 2.99	0.09
placental + umbilical cord weight, g	584.23 ± 107.9 (26)	680.11 ± 114.4 (9)	0.51	653.53 ± 198.7 (15)	0.67	906.33 ± 164.5	0.00

Data are shown as mean ± standard deviation (SD) or percentage (absolute numbers). Absolute numbers are shown in parentheses if the characteristic was not available for all participants of the group. CTL = healthy controls; GDM = gestational diabetes mellitus; DM1 = type 1 diabetes mellitus; DM2 = type 2 diabetes mellitus; NA = not available; BMI = body mass index; SBP = systolic blood pressure; DBP = diastolic blood pressure; HbA1c = hemoglobin A1c. Comparison of a single group to CTL was assessed by one-way ANOVA with multiple comparisons.

**Table 2:** Clinical characteristics of study group participants, including 29 CTLs and DM superimposed by PE (*n* = 16).

Characteristics	CTL (*n* = 29)	GDM + PE (*n* = 4)	*p*-Value CTL vs. GDM + PE	DM1 + PE (*n* = 8)	*p*-Value CTL vs. DM1 + PE	DM2 + PE (*n* = 4)	*p*-Value CTL vs. DM2 + PE
**maternal characteristics <20 weeks gestation**							
BMI	23.68 ± 4.06	31.91 ± 4.47	0.00	24.68 ± 2.85	0.99	28.07 ± 5.69	0.30
SBP, mmHg	111.03 ± 9.41	118.5 ± 11.21	0.82	125.88 ± 16.3	0.02	124 ± 29.02	0.25
DBP, mmHg	66.21 ± 7.04	76.25 ± 8.96	0.08	72.25 ± 6.5	0.25	69.25 ± 16.4	0.97
HbA1c 2. Trimester, %	NA	5.5 ± 0.1 (2)	NA	6.63 ± 0.66	NA	5.67 ± 0.12 (2)	NA
smoking, % (n)	13.79 (4)	25 (1)	NA	25 (2)	NA	0 (0)	NA
height, m	1.70 ± 0.06	1.61 ± 0.11	0.06	1.69 ± 0.04	>0.99	1.57 ± 0.14	0.00
weight, kg	68.17 ± 11.96	83.5 ± 20.76	0.16	68.75 ± 7.15	>0.99	70.25 ± 20.76	0.99
**maternal characteristics at delivery**							
BMI at delivery	28.79 ± 3.98	38.05 ± 3.55	0.00	32.07 ± 3.65	0.39	31.54 ± 7.39	0.85
pre-operative SBP, mmHg	124.42 ± 12.22 (26)	153 ± 16.92 (3)	0.03	169.38 ± 25.05	0.00	152.67 ± 13.65 (3)	0.02
pre-operative DBP, mmHg	72.96 ± 10.78 (26)	90.33 ± 8.51 (3)	0.08	99.13 ± 10.91	0.00	88.0 ± 8.66 (3)	0.17
proteinuria, % (n)	4 (1)	100 (4)	NA	100 (8)	NA	100 (4)	NA
HbA1c 3. Trimester, %	NA	6.25 ± 0.05 (2)	NA	6.6 ± 0.56	NA	6.65 ± 0.25 (2)	NA
medication, % (n) [insulin, metformin]	0 (0)	25 (1)	NA	100 (8)	NA	100 (4)	NA
gestational age, days	271.93 ± 7.94	247.25 ± 18.77	0.00	248.38 ± 13.75	0.00	249.25 ± 31.10	0.02
age, years	32.66± 4.50	29.0 ± 4.08	0.57	32.13 ± 6.11	0.98	34.25 ± 5.38	0.98
parity, count	0.93 ± 0.88	0.75 ± 0.50	0.99	0.5 ± 0.76	0.70	0.5 ± 1.00	0.90
gravidity, count	2.72 ± 1.25	1.75 ± 0.50	0.60	2.0 ± 1.07	0.60	1.75 ± 1.50	0.60
blood sugar, mmol/l	3.98 ± 0.48 (9)	4.28 ± 0.50	0.99	6.32 ± 1.8 (7)	0.00	3.9 ± 0.30 (2)	>0.99
**birth outcome**							
fetal sex, female/male, count	15/14	3/1	NA	4/4	NA	3/1	NA
birth weight, g	3342 ± 450	2422 ± 671	0.17	3305 ± 830	>0.99	2989 ± 1845	0.95
birth length, cm	50.05 ± 1.49 (21)	46.75 ± 2.06	0.33	48.8 ± 0.84 (5)	0.96	38 ± 11.30 (2)	0.00
percentile birthweight	51.04 ± 27.68	28.29 ± 26.60	0.68	76.97 ± 31.06	0.21	51.6 ± 55.17	>0.99
placental + umbilical cord weight, g	584.23 ± 107.9 (26)	494.25 ± 111.7	0.86	615.75 ± 201.3	0.99	577.75 ± 218.0	>0.99

Data are shown as mean ± standard deviation (SD) or percentage (absolute number). Total numbers are shown in parentheses if the characteristic was not available for all participants of the group. CTL = healthy controls; GDM + PE = gestational diabetes mellitus superimposed with preeclampsia; DM1 + PE = type 1 diabetes mellitus superimposed with preeclampsia; DM2 + PE = type 2 diabetes mellitus superimposed with preeclampsia; NA = not available; BMI = body mass index; SBP = systolic blood pressure; DBP = diastolic blood pressure; HbA1c = haemoglobin A1c. Comparison of a single group to CTL was assessed by one-way ANOVA with multiple comparisons.

**Table 3:** Clinical characteristics of study groups with all diabetes subgroups combined (*n* = 77).

	CTL (*n* = 29)	Diabetes (*n* = 32)	*p*-Value CTL vs. Diabetes	Diabetes + PE (*n* = 16)	*p*-Value CTL vs. Diabetes + PE
**maternal characteristics <20 weeks gestation**					
BMI	23.68 ± 4.06	27.15 ± 5.21	0.01	27.33 ± 4.87	0.03
SBP, mmHg (*n*)	111.03 ± 9.41	114.87 ± 10.47 (31)	0.40	123.56 ± 18.09	0.00
DBP, mmHg (*n*)	66.21 ± 7.04	70.68 ± 6.28 (31)	0.04	72.50 ± 9.81	0.02
HbA1c 2. Trimester, % (*n*)	NA	5.95 ± 0.69 (22)	NA	6.23 ± 0.73 (13)	NA
smoking, % (*n*)	13.79 (4)	12.50 (4)	NA	18.75 (3)	NA
height, m	1.70 ± 0.06	1.76 ± 0.05	0.33	1.64 ± 0.10	0.01
weight, kg	68.17 ± 11.96	76.03 ± 14.58	0.06	72.81 ± 15.40	0.49
**maternal characteristics at delivery**					
BMI at delivery	28.79 ± 3.98	31.80 ± 5.69 (31)	0.04	33.43 ± 5.22	0.00
pre-operative SBP, mmHg	124.42 ± 12.22 (26)	136.96 ± 13.02 (26)	0.00	161.93 ± 22.15 (14)	0.00
pre-operative DBP, mmHg	72.96 ± 10.78 (26)	79.12 ± 11.66 (26)	0.09	94.86 ± 10.65 (14)	0.00
Proteinuria, % (*n*)	4 (1)	6.25 (2)	NA	100 (16)	NA
HbA1c 3. Trimester, %	NA	6.08 ± 0.58 (29)	NA	6.55 ± 0.49 (12)	NA
medication metformin, % (*n*)	0 (0)	9.38 (3)	NA	18.75 (3)	NA
insulin, % (*n*)	0 (0)	68.75 (22)	NA	68.75 (11)	NA
early-onset PE % (*n*)	0 (0)	0 (0)	NA	37.5 (6)	NA
age, years	32.66± 4.50	33.72 ± 3.96	0.59	31.88 ± 5.50	0.82
parity, count	0.93 ± 0.88	0.81 ± 0.74	0.81	0.56 ± 0.72	0.26
gravidity, count	2.72 ± 1.25	2.63 ± 1.31	0.94	1.88 ± 1.03	0.06
blood sugar, mmol/l	3.98 ± 0.48 (9)	5.92 ± 1.59 (25)	0.00	5.32 ± 1.74 (13)	0.08
**birth outcome**					
fetal sex, female/male, count	15/14	16/16	NA	10/6	NA
birth weight, g	3342 ± 450	3732 ± 863 (31)	0.12	3005 ± 1110	0.32
birth length, cm	50.05 ± 1.49 (21)	50.39 ± 3.87 (27)	0.94	46.09 ± 5.59 (11)	0.01
percentile birthweight	51.04 ± 27.68	78.44 ± 30.31 (31)	0.00	58.46 ± 40.48	0.70
placental + umbilical cord weight, g	584.23 ± 107.9 (26)	690.48 ± 183.2 (27)	0.03	575.88 ± 183.1	0.98

Data are shown as mean ± standard deviation or percentage (total number). Absolute numbers are shown in parentheses if the characteristic was not available for all participants of the group. CTL = healthy controls; Diabetes = includes patients with DM1, DM2 and GDM; Diabetes + PE = includes patients with diabetes superimposed with preeclampsia (DM1 + PE, DM2 + PE, GDM + PE); NA = not available; BMI = body mass index; SBP = systolic blood pressure; DBP = diastolic blood pressure; HbA1c = hemoglobin A1c. Comparison of one group to healthy controls was performed by one-way ANOVA with multiple comparisons.

## Data Availability

The data sets generated during and/or analyzed during the current study are available from the corresponding authors on reasonable request.

## Supplementary Materials

The following supporting information can be downloaded at: https://www.mdpi.com/article/10.3390/ijms232315388/s1; Supplementary Table S1, Supplementary Table S2.
