# Supplementary material for: Placental Transcriptome Profiling in Subtypes of Diabetic Pregnancies Is Strongly Confounded by Fetal Sex
Source: Int J Mol Sci. 2022 Dec 6;23(23):15388. doi: 10.3390/ijms232315388 (PMC9740420; doi:10.3390/ijms232315388)
Supplement: Supplementary file 1 [file ijms-23-15388-s001.zip › ijms-2021835-supplementary.pdf]

Supplemental material

Supplement Table S1. Differential expressed genes in diabetic and healthy placentas.

Comparison	Gene iD	Gene Name	Base Mean	log2fc	Adjusted p-value
DM2 vs CTL, up-regulated	ENSG00000108669	CYTH1	830	0.31	0.035
	ENSG00000170145	SIK2	610	0.31	0.026
	ENSG00000118689	FOXO3	1200	0.32	0.043
	ENSG00000146039	SLC17A4	2.9	0.42	0.02
	ENSG00000124098	FAM210B	450	0.44	0.026
	ENSG00000148842	CNNM2	570	0.46	0.014
	ENSG00000174306	ZHX3	400	0.46	0.042
	ENSG00000180667	YOD1	530	0.47	0.045
	ENSG00000198876	DCAF12	870	0.47	0.019
	ENSG00000153253	SCN3A	13	0.49	0.009
	ENSG00000011478	QPCTL	39	0.5	0.05
	ENSG00000167916	KRT24	4.1	0.5	0.00048
	ENSG00000171608	PIK3CD	170	0.52	0.044
	ENSG00000119636	BBOF1	31	0.55	0.045
	ENSG00000137198	GMPR	67	0.55	0.026
	ENSG00000197465	GYPE	3.3	0.55	0.043
	ENSG00000013306	SLC25A39	610	0.56	0.011
	ENSG00000143416	SELENBP1	240	0.56	0.043
	ENSG00000066926	FECH	500	0.57	0.009
	ENSG00000187997	C17orf99	1.6	0.57	0.019
	ENSG00000198838	RYR3	15	0.57	0.019
	ENSG00000198858	R3HDM4	210	0.57	0.037
	ENSG00000204613	TRIM10	2.3	0.57	0.01
	ENSG00000119138	KLF9	150	0.58	0.048
	ENSG00000165702	GFI1B	4.8	0.58	0.033
	ENSG00000166741	NNMT	350	0.58	0.041
	ENSG00000213626	LBH	590	0.58	0.048

	ENSG00000256269	HMBS	75	0.59	0.03
	ENSG00000168386	FILIP1L	630	0.6	0.038
	ENSG00000197405	C5AR1	87	0.6	0.035
	ENSG00000157103	SLC6A1	20	0.61	0.042
	ENSG00000162426	SLC45A1	2.6	0.61	0.026
	ENSG00000188536	HBA2	3200	0.61	0.04
	ENSG00000272808	NA	5.8	0.61	0.019
	ENSG00000104856	RELB	64	0.63	0.0084
	ENSG00000136542	GALNT5	2.8	0.63	0.009
	ENSG00000166947	EPB42	54	0.63	0.021
	ENSG00000113070	HBEGF	82	0.64	0.027
	ENSG00000137801	THBS1	5700	0.64	0.026
	ENSG00000158578	ALAS2	620	0.64	0.022
	ENSG00000011422	PLAUR	83	0.65	0.011
	ENSG00000042062	RIPOR3	55	0.65	0.014
	ENSG00000125398	SOX9	3.2	0.65	0.00063
	ENSG00000173432	SAA1	29	0.65	0.00016
	ENSG00000280237	MIR4697HG	38	0.65	0.024
	ENSG00000206172	HBA1	590	0.66	0.019
	ENSG00000250361	GYPB	13	0.66	0.009
	ENSG00000164949	GEM	33	0.67	0.017
	ENSG00000196565	HBG2	11000	0.67	0.01
	ENSG00000206177	HBM	18	0.67	0.009
	ENSG00000136929	HEMGN	140	0.68	0.011
	ENSG00000147454	SLC25A37	900	0.68	0.009
	ENSG00000178878	APOLD1	910	0.69	0.009
	ENSG00000211582	MIR758	22	0.69	0.0067
	ENSG00000243444	PALM2	41	0.69	0.009
	ENSG00000184792	OSBP2	74	0.7	0.009
	ENSG00000198336	MYL4	26	0.7	0.0098
	ENSG00000047597	XK	52	0.71	0.009
	ENSG00000170180	GYPA	30	0.72	0.0031
	ENSG00000130775	THEMIS2	150	0.73	0.0022

	ENSG00000075340	ADD2	8.7	0.74	0.0017
	ENSG00000106327	TFR2	5.1	0.74	0.0019
	ENSG00000004939	SLC4A1	400	0.76	0.0015
	ENSG00000188672	RHCE	10	0.76	0.0028
	ENSG00000163554	SPTA1	150	0.77	0.0022
	ENSG00000125845	BMP2	88	0.78	9e-04
	ENSG00000187010	RHD	4.7	0.79	0.00011
	ENSG00000184557	SOCS3	420	0.81	0.00016
	ENSG00000029534	ANK1	110	0.82	0.00075
	ENSG00000169507	SLC38A11	26	0.82	0.00046
	ENSG00000196843	ARID5A	130	0.84	0.00011
	ENSG00000112077	RHAG	9.6	0.85	2.1e-05
	ENSG00000164398	ACSL6	9.2	0.85	0.00017
	ENSG00000169877	AHSP	47	0.85	0.00016
	ENSG00000250182	NA	24	1.3	5.7e-11
DM2 vs CTL, down-regulated	ENSG00000198711	SSBP3-AS1	14	-0.59	0.048
	ENSG00000273035	NA	45	-0.55	0.036
	ENSG00000204941	PSG5	29000	-0.53	0.048
	ENSG00000178966	RMI1	140	-0.47	0.043
	ENSG00000180346	TIGD2	790	-0.46	0.019
	ENSG00000171943	SRGAP2C	260	-0.45	0.029
	ENSG00000159596	TMEM69	160	-0.44	0.009
	ENSG00000030419	IKZF2	880	-0.38	0.042
	ENSG00000157259	GATAD1	730	-0.34	0.021
	ENSG00000155313	USP25	2300	-0.33	0.036
	ENSG00000165516	KLHDC2	580	-0.33	0.027
	ENSG00000197323	TRIM33	2900	-0.3	0.0053
	ENSG00000141446	ESCO1	680	-0.27	0.038
	ENSG00000000457	SCYL3	530	-0.26	0.038
	ENSG00000153147	SMARCA5	2100	-0.26	0.042
	ENSG00000092439	TRPM7	2700	-0.25	0.043
	ENSG00000005810	MYCBP2	3900	-0.21	0.021
	ENSG00000116560	SFPQ	5800	-0.19	0.038

GDM vs DM2, down-regulated	ENSG00000250182	NA	24	-1.1	1.3e-06
	ENSG00000169507	SLC38A11	26	-0.96	1.3e-05
	ENSG00000196843	ARID5A	130	-0.83	0.00064
	ENSG00000088320	REM1	15	-0.78	0.0051
	ENSG00000042062	RIPOR3	55	-0.76	0.0076
	ENSG00000256269	HMBS	75	-0.74	0.0078
	ENSG00000184557	SOCS3	420	-0.72	0.009
	ENSG00000163554	SPTA1	150	-0.71	0.026
	ENSG00000206172	HBA1	590	-0.7	0.025
	ENSG00000243444	PALM2	41	-0.68	0.041
	ENSG00000105650	PDE4C	36	-0.66	0.041
	ENSG00000105650	LOC729966	36	-0.66	0.041
	ENSG00000143416	SELENBP1	240	-0.66	0.031
	ENSG00000164398	ACSL6	9.2	-0.65	0.032
	ENSG00000188536	HBA2	3200	-0.65	0.043
	ENSG00000213626	LBH	590	-0.65	0.046
	ENSG00000148842	CNNM2	570	-0.63	0.00056
	ENSG00000169877	AHSP	47	-0.63	0.046
	ENSG00000013306	SLC25A39	610	-0.62	0.012
	ENSG00000017483	SLC38A5	5.2	-0.62	0.046
	ENSG00000124098	FAM210B	450	-0.59	0.0018
	ENSG00000198876	DCAF12	870	-0.54	0.02
	ENSG00000153253	SCN3A	13	-0.49	0.0025
GDM vs DM2, up-regulated	ENSG00000109046	WSB1	8400	0.29	0.046
	ENSG00000165516	KLHDC2	580	0.36	0.043
	ENSG00000265185	SNORD3B-1	160	0.38	0.012
	ENSG00000134245	WNT2B	450	0.62	0.024
DM2 vs DM1, up-regulated	ENSG00000173432	SAA1	29	0.56	0.0049
	ENSG00000196843	ARID5A	130	0.69	0.038
	ENSG00000169507	SLC38A11	26	0.94	3.8e-05
	ENSG00000250182	NA	24	1.3	6.4e-10
GDM vs DM1, up-regulated	ENSG00000181019	NQO1	63	0.53	0.025
	ENSG00000179256	SMCO3	11	0.82	0.012

PE vs healthy	ENSG00000229344	NA	11	0.34	0.013

Supplement Table S2. Differential expressed genes between placentas with male vs placenta with female foetuses.

Comparison	Gene iD	Gene Name	Base Mean	log2fc	Adjusted p-value
Male vs female up-regulated	ENSG00000001626	CFTR	40.2	0.375	0.000837
	ENSG00000012817	KDM5D	643	6.36	0
	ENSG00000047634	SCML1	344	0.241	0.0342
	ENSG00000067048	DDX3Y	931	6	0
	ENSG00000067646	ZFY	323	5.4	0
	ENSG00000092377	TBL1Y	4.25	1.28	3.78e-55
	ENSG00000099715	PCDH11Y	389	3.94	0
	ENSG00000099725	PRKY	186	5.02	0
	ENSG00000102271	KLHL4	3.57	0.239	0.0492
	ENSG00000113649	TCERG1	1140	0.0794	0.00876
	ENSG00000114374	USP9Y	889	6.17	0
	ENSG00000118785	SPP1	8530	0.327	0.0198
	ENSG00000121741	ZMYM2	1860	0.134	0.00864
	ENSG00000129824	RPS4Y1	576	6.49	0
	ENSG00000131002	TXLNGY	251	4.4	0
	ENSG00000143153	ATP1B1	338	0.277	0.00205
	ENSG00000147180	ZNF711	136	0.299	0.0334
	ENSG00000151136	BTBD11	42.9	0.313	0.0336
	ENSG00000154620	TMSB4Y	7.03	1.91	8.93e-114
	ENSG00000165138	ANKS6	177	0.201	0.00918
	ENSG00000165246	NLGN4Y	23.2	0.615	7.7e-20
	ENSG00000165259	HDX	11.6	0.338	0.0111
	ENSG00000172294	CSPG4P4Y	0.653	0.141	0.0118
	ENSG00000176728	TTTY14	40.4	3.1	0
	ENSG00000183778	B3GALT5	28.7	0.327	0.0229
	ENSG00000183878	UTY	1020	6.75	0
	ENSG00000185070	FLRT2	42.8	0.309	0.0429
	ENSG00000185275	CD24P4	2.88	0.873	5.75e-28

	ENSG00000186453	FAM228A	12.7	0.318	0.0328
	ENSG00000188399	ANKRD36P1	2.93	0.181	0.000858
	ENSG00000189369	GSPT2	47.2	0.295	0.0455
	ENSG00000198692	EIF1AY	131	4.82	0
	ENSG00000206159	GYG2P1	26.2	3.02	1.92e-296
	ENSG00000214207	KRT18P10	12.1	2.05	2.69e-133
	ENSG00000215506	TPTE2P4	4.93	0.164	0.00273
	ENSG00000215548	FRG1JP	36.8	0.55	1.59e-8
	ENSG00000215580	BCORP1	0.544	0.208	0.000346
	ENSG00000219607	PPP1R3G	18.2	0.27	0.0401
	ENSG00000219665	CTD-2006C1.2	84.8	0.211	0.0134
	ENSG00000224060	ARSEP1	0.623	0.18	0.00121
	ENSG00000225117	ARSDP1	1.94	0.527	1.68e-15
	ENSG00000226555	AGKP1	2.91	0.374	5.6e-10
	ENSG00000226863	SHROOM2P1	1.08	0.457	1.59e-12
	ENSG00000227447	XGY1	0.935	0.306	1.7e-7
	ENSG00000228764	ZNF885P	0.694	0.224	0.0000589
	ENSG00000228786	LINC00266-4P	1.68	0.519	4.73e-15
	ENSG00000229163	NAP1L1P2	1.22	0.472	4.59e-13
	ENSG00000229236	TTTY10	14	2.63	1.91e-222
	ENSG00000229238	PPP1R12BP1	2.43	0.164	0.00727
	ENSG00000229308	AC010084.1	9.83	2.03	4.36e-128
	ENSG00000230663	FAM224B	2.66	0.89	1.81e-31
	ENSG00000231535	LINC00278	46.1	3.42	0
	ENSG00000232195	TOMM22P2	0.427	0.171	0.00908
	ENSG00000232226	ARSFP1	0.533	0.179	0.00195
	ENSG00000232730	FAM8A4P	1.35	0.463	7.06e-13
	ENSG00000233070	ZFY-AS1	4.72	1.35	4.82e-61
	ENSG00000233864	TTTY15	208	5.38	0
	ENSG00000234511	C5orf58	5.29	0.278	0.0484
	ENSG00000235462	TAB3P1	3.23	1.05	3.33e-40
	ENSG00000235649	MXRA5Y	6.37	0.265	0.0439
	ENSG00000237659	RNASEH2CP1	2.44	0.589	4.59e-18

	ENSG00000241859	ANOS2P	17.4	2.43	3.2e-184
	ENSG00000251841	RNU6-1334P	0.764	0.256	0.00000708
	ENSG00000251996	Y_RNA	0.804	0.359	4.64e-9
	ENSG00000252468	RNU2-57P	5.4	1.59	3.25e-80
	ENSG00000252766	RNU6-255P	1.13	0.498	6.69e-14
	ENSG00000257599	OVCH1-AS1	1.67	0.208	0.00551
	ENSG00000260197	RP11-424G14.1	8.33	2	1.39e-123
	ENSG00000267793	RP11-576C2.1	3.81	1.1	8.68e-44
	ENSG00000273906	RP11-115H13.1	3.56	1.13	3.29e-45
	ENSG00000277146	RP11-557B9.1	1.2	0.123	0.0322
	ENSG00000277438	RP11-256K9.1	2.03	0.878	2.77e-30
	ENSG00000278212	MAFIP	7.34	1.19	6.62e-46
	ENSG00000278847	RP11-414C23.1	1.5	0.664	2.36e-20
	ENSG00000282870	FRG1DP	83.3	0.289	0.00481
	ENSG00000282995	FRG1EP	50.1	0.342	0.00727
	ENSG00000283023	FRG1GP	114	0.266	0.0107
	ENSG00000283047	FRG1FP	9.56	0.463	0.000011
Male vs female down-regulated	ENSG00000001497	LAS1L	443	-0.202	5.19e-10
	ENSG00000005889	ZFX	2050	-0.484	1.87e-67
	ENSG00000012174	MBTPS2	527	-0.402	1.89e-15
	ENSG00000018610	CXorf56	582	-0.2	0.0226
	ENSG00000072501	SMC1A	2130	-0.543	1.13e-137
	ENSG00000072506	HSD17B10	242	-0.298	2.45e-7
	ENSG00000086712	TXLNG	227	-0.191	7.73e-7
	ENSG00000089682	RBM41	400	-0.146	0.0143
	ENSG00000101846	STS	10100	-0.632	5.67e-19
	ENSG00000101882	NKAP	246	-0.182	0.00000357
	ENSG00000101966	XIAP	1340	-0.113	0.00481
	ENSG00000101972	STAG2	2690	-0.118	0.0401
	ENSG00000102030	NAA10	315	-0.326	2.3e-13
	ENSG00000102081	FMR1	2850	-0.146	0.0231
	ENSG00000102225	CDK16	999	-0.263	1.47e-11
	ENSG00000102309	PIN4	158	-0.24	0.0000269

	ENSG00000115808	STRN	1130	-0.113	0.0289
	ENSG00000122824	NUDT10	2.71	-0.19	0.0021
	ENSG00000122861	PLAU	1360	-0.242	0.0492
	ENSG00000123496	IL13RA2	19	-0.315	0.00346
	ENSG00000124486	USP9X	12600	-0.179	0.0376
	ENSG00000125676	THOC2	1690	-0.135	0.000363
	ENSG00000126012	KDM5C	2830	-0.642	6.54e-117
	ENSG00000130021	PUDP	413	-0.67	4.69e-31
	ENSG00000130741	EIF2S3	1180	-0.395	8.88e-50
	ENSG00000130985	UBA1	2090	-0.323	2.74e-32
	ENSG00000133789	SWAP70	1960	-0.123	0.0289
	ENSG00000143365	RORC	6.83	-0.317	0.0261
	ENSG00000147050	KDM6A	1370	-0.798	1.11e-149
	ENSG00000147065	MSN	2350	-0.152	0.0492
	ENSG00000147099	HDAC8	317	-0.308	3.64e-17
	ENSG00000147113	CXorf36	1290	-0.521	7.61e-16
	ENSG00000147162	OGT	4760	-0.151	0.00198
	ENSG00000154162	CDH12	15.6	-0.189	0.0385
	ENSG00000165195	PIGA	534	-0.21	0.0401
	ENSG00000165775	FUNDC2	406	-0.126	0.0492
	ENSG00000166473	PKD1L2	159	-0.31	0.0395
	ENSG00000169239	CA5B	141	-0.242	0.000607
	ENSG00000169249	ZRSR2	110	-0.275	3.14e-7
	ENSG00000172943	PHF8	973	-0.133	0.000288
	ENSG00000173674	EIF1AX	642	-0.555	2.51e-51
	ENSG00000173681	CXorf23	536	-0.375	1.42e-14
	ENSG00000181704	YIPF6	1850	-0.23	0.000264
	ENSG00000185515	BRCC3	290	-0.188	0.000346
	ENSG00000186312	CA5BP1	123	-0.249	0.0000215
	ENSG00000188419	CHM	459	-0.337	1.85e-27
	ENSG00000198034	RPS4X	3520	-0.395	4.09e-21
	ENSG00000200354	SNORA71D	53	-0.28	0.031
	ENSG00000200792	SNORA80A	39.4	-0.294	0.0303

	ENSG00000207088	SNORA7B	134	-0.219	0.0312
	ENSG00000208892	SNORA49	361	-0.24	0.00198
	ENSG00000210196	MT-TP	19.1	-0.307	0.0339
	ENSG00000215301	DDX3X	8070	-0.557	1.4e-74
	ENSG00000222489	SNORA79	183	-0.23	0.0308
	ENSG00000223546	LINC00630	213	-0.184	0.00732
	ENSG00000224430	MKRN5P	348	-0.171	0.0292
	ENSG00000224975	INE1	73.5	-0.362	2.85e-8
	ENSG00000225091	SNORA71A	66.7	-0.28	0.0226
	ENSG00000225470	JPX	438	-0.365	2.13e-29
	ENSG00000226641	RP13-13A3.1	4.67	-0.388	0.00109
	ENSG00000227329	RP11-258C19.4	0.77	-0.223	0.00921
	ENSG00000228550	RP11-483M24.2	6.27	-0.348	0.00918
	ENSG00000228906	RP13-216E22.4	72	-0.259	0.00375
	ENSG00000229807	XIST	13000	-0.751	4.77e-24
	ENSG00000230590	FTX	1940	-0.142	0.0144
	ENSG00000230797	YY2	31.8	-0.261	0.014
	ENSG00000235262	KDM5C-IT1	17.9	-0.591	7.37e-13
	ENSG00000236120	RP11-733O18.1	29.4	-0.314	0.0222
	ENSG00000239407	LL0XNC01-237H1.2	91.4	-0.179	0.0339
	ENSG00000252481	SCARNA13	1750	-0.162	0.0133
	ENSG00000265727	RN7SL648P	257	-0.161	0.00195
	ENSG00000270641	TSIX	2.62	-0.763	2.48e-21
	ENSG00000271430	RP3-368A4.5	1820	-0.133	0.0251
	ENSG00000271533	RP3-368A4.6	1560	-0.149	0.0206
	ENSG00000272533	SNORA28	31.8	-0.278	0.00581
	ENSG00000279682	RP11-791N19.1	16.6	-0.479	0.00000208
